# Trial of Optimal Personalised Care After Treatment for Gynaecological cancer (TOPCAT-G): a study protocol for a randomised feasibility trial

**DOI:** 10.1186/s40814-016-0108-5

**Published:** 2016-11-23

**Authors:** Kirstie Pye, Nicola Totton, Nicholas Stuart, Rhiannon Whitaker, Val Morrison, Rhiannon Tudor Edwards, Seow Tien Yeo, Laura J. Timmis, Caryl Butterworth, Liz Hall, Tekendra Rai, Zoe Hoare, Richard D. Neal, Clare Wilkinson, Simon Leeson

**Affiliations:** 1 North Wales Organisation for Randomised Trials in Health (NWORTH), IMSCaR, COHABS, Bangor University, Bangor, UK; 2 Betsi Cadwaladr University Health Board, Bangor, UK; 3 Whitaker Research Ltd., Bangor, UK; 4 School of Psychology, COHABS, Bangor University, Bangor, UK; 5 Centre for Health Economics and Medicines Evaluation, IMSCaR, COHABS, Bangor University, Bangor, UK; 6 Leeds Institute of Health Sciences, University of Leeds, Leeds, UK; 7 North Wales Centre for Primary Care Research, COHABS, Bangor University, Bangor, UK

**Keywords:** Gynaecological cancer follow-up, Nurse-led telephone intervention, Feasibility, Randomised controlled trial, Quality of life

## Abstract

**Background:**

Gynaecological cancers are diagnosed in over 1000 women in Wales every year. We estimate that this is costing the National Health Service (NHS) in excess of £1 million per annum for routine follow-up appointments alone. Follow-up care is not evidence-based, and there are no definitive guidelines from The National Institute for Health and Care Excellence (NICE) for the type of follow-up that should be delivered. Standard care is to provide a regular medical review of the patient in a hospital-based outpatient clinic for a minimum of 5 years. This study is to evaluate the feasibility of a proposed alternative where the patients are delivered a specialist nurse-led telephone intervention known as Optimal Personalised Care After Treatment for Gynaecological cancer (OPCAT-G), which comprised of a protocol-based patient education, patient empowerment and structured needs assessment.

**Methods:**

The study will recruit female patients who have completed treatment for cervical, endometrial, epithelial ovarian or vulval cancer within the previous 3 months in Betsi Cadwaladr University Health Board (BCUHB) in North Wales. Following recruitment, participants will be randomised to one of two arms in the trial (standard care or OPCAT-G intervention). The primary outcomes for the trial are patient recruitment and attrition rates, and the secondary outcomes are quality of life, health status and capability, using the EORTC QLQ-C30, EQ-5D-3L and ICECAP-A measures. Additionally, a client service receipt inventory (CSRI) will be collected in order to pilot an economic evaluation.

**Discussion:**

The results from this feasibility study will be used to inform a fully powered randomised controlled trial to evaluate the difference between standard care and the OPCAT-G intervention.

**Trial registration:**

ISRCTN45565436.

## Background

In the UK, gynaecological cancers including endometrial (c. 8500 per year), ovarian (c. 7000 per year), cervical (c. 3000 per year) and vulval (c. 1200 per year) cancer are retrospectively the fourth, fifth, twelfth, and twentieth most common cancer sites in women [1–4]. Over 1000 females are diagnosed and treated with gynaecological cancers each year in Wales (ovarian = 365, uterine = 539, cervical = 164) [5]. Three North Wales hospitals comprising Betsi Cadwaladr University Health Board (BCUHB) (Ysbyty Gwynedd, Ysbyty Glan Clwyd, and Ysbyty Maelor) serve a population of approximately 678,000 people [6], with 92 ovarian cancer, 107 endometrial cancer and 35 cervical cancer newly diagnosed in 2014 [7].

### Aims of follow-up

The aims of follow-up care include the management of patients’ physical and psychological morbidity and the prevention or early detection of local recurrence, distant metastases or appearance of second cancers [8, 9]. Follow-up care is routine for women who have completed treatment for gynaecological cancer but is not evidence-based. Currently, there is no agreement as to what follow-up care is effective, and there are no guidelines from The National Institute for Health and Care Excellence (NICE) as to what form or frequency of follow-up is appropriate after treatment for gynaecological cancer.

### Standard approach to follow-up

Following completion of treatment for gynaecological cancer, the traditional medical practice is to regularly review the patient in a hospital-based, outpatient clinic over a period of years. These regular reviews occur according to a locally or regionally agreed schedule for at least 5 years after completion of treatment. In addition to clinical review, patients may have blood tests, scans or other tests as required. Multiple unproven assumptions lie behind this standard approach to gynaecological cancer follow-up; one being that it will benefit the patient by detecting recurrence early, thus allowing effective treatment and improving survival, or alternatively will offer reassurance to the patient that all is well. There is however little evidence to support this approach. In general, early detection of recurrence is unlikely to improve survival if there is no intervention capable of producing a cure [10]. For the majority of gynaecological cancers, any treatment for recurrence is palliative and not curative. Whilst a few retrospective studies suggest that survival was improved when recurrent cervical or endometrial cancer was detected at routine follow-up rather than when symptoms develop [11–13], the majority of patients relapse with symptoms that would prompt reassessment even if the patient was not on routine review [14–17]. A randomised controlled trial (RCT) in ovarian cancer that assessed the value of detecting relapse early by using serum CA125 measurements as part of routine follow-up confirmed that whilst the test was able to detect pre-symptomatic recurrence, this did not lead to any survival benefit. It did however lead to an inferior quality of life due to further chemotherapy provision and more treatment-related morbidity [18]. This study again raises doubts about the value of detecting pre-symptomatic recurrence.

Patients may also receive false reassurance (when follow-up finds ‘no symptoms’) or they may wait for their next routine appointment to disclose symptoms [19] thus possibly delaying detection and appropriate symptom management. Furthermore, there is evidence that hospital-based, medical follow-up fails to address many of the broader needs that patients have following treatment for gynaecological cancers. Patients commonly report problems with anxiety and depression [20] and reduced health-related quality of life (QoL) [21], with specific problems including the physical effects of treatment such as distressing disturbance of bowel and bladder function, sexual relations and relationships, psychological health and employment problems [22, 23]. These issues are reflected in studies of patient-expressed needs. Female cancer patients tend to express more needs than male cancer patients and report less satisfaction with support generally [24, 25]. Gynaecological cancer patients have reported a high level of need for detailed information, information regarding how best to recover from surgery or to avoid recurrence and information regarding physiological and psychological issues [24, 26, 27]. There is evidence that doctors in routine follow-up clinics often fail to identify such problems and, if they do, they fail to address them effectively [28, 29].

Finally, the standard approach to follow-up burdens the health service with significant costs and challenges. The increasing numbers of patients who have received treatment for gynaecological cancer in conjunction with rising survival rates mean that the number of patients on follow-up is rising. A significant amount of medical, nursing and administrative time is spent on arranging and undertaking potentially unnecessary medical reviews. The unit cost for a non-admitted consultant-led, face-to-face, follow-up appointment in gynaecology oncology for the year 2014–2015 was £168 [30]. We estimate that in Wales, the National Health Service (NHS) cost of these routine follow-up appointments, excluding the cost of any tests ordered, is in excess of £1 million per annum based on current survival rates and national follow-up policies. This is in addition to any societal costs such as time taken from work to attend clinics and additional childcare costs.

### Alternative approaches to follow-up

In response to these challenges, a range of additional, alternative or supplementary approaches have been developed. These include risk-stratified care pathways, nurse-led clinics, telephone follow-up, reduced frequency of follow-up and remote surveillance strategies where biomarker or radiological follow-up is key. The National Cancer Survivorship Initiative [31] recommends a stratified approach to cancer follow-up care as opposed to the current traditional pathway. The NHS in England is piloting this risk-stratified approach that divides cancer patients into three groups: complex and requiring continued hospital care; shared care, for patients that require hospital care supplemented by education and support; and a self-care group that can be discharged from routine hospital follow-up [32]. However, the criteria for these groups are not yet fully defined and neither is the proportion of patients that will fall into each. Furthermore, there is as yet no evidence that this approach is superior to standard care or is the best alternative. We have recently undertaken a national survey of clinical practice in follow-up in all gynaecological cancers [33] which reported that a wide range of follow-up approaches are used across the UK despite there being little evidence that they lead to better outcomes for patients or are more cost-effective. Although the networks reported a wide range of practice in relation to tests and frequency of review, the survey confirmed that hospital-based, medical review remains the standard approach. A minority used nurse-led or telephone follow-up, and general practitioners (GPs) were rarely involved in routine care.

We propose an approach that aims to provide nurse-led telephone follow-up care for patients. Investigations into patterns of post-treatment cancer follow-up should assess recovery from treatment toxicity and social and psychological rehabilitation as well as health economics and the detection of relapse. Care should also be individualised so that patients can have the information they need as well as having their individual problems identified and managed. There is a pressing need for the NHS to develop patterns of care for patients who have had treatment for cancer that will address their needs, maximise their QoL and allow timely diagnosis of relapse yet uses NHS resources in a cost-effective manner. It is also important to convince patients, doctors and health service managers that this new approach can be adopted without any adverse effect on survival of patients.

### Purpose and scope

This study will evaluate the feasibility of a nurse-led needs assessment in the gynaecology cancer setting in North Wales and its utility to health care professionals when making decisions about supportive care, compared to disease- and site-matched patients not receiving the nurse-led needs assessment. The nurse-led intervention will be known as Optimal Personalised Care After Treatment for Gynaecological cancer (OPCAT-G) and will comprise patient education, patient empowerment and protocol-based, structured needs assessment undertaken with Gynae Oncology clinical nurse specialists (CNSs) by telephone. Patients will receive an initial structured telephone interview with a gynaecological CNS and a booklet including information on symptoms of recurrence and possible long-term physical and psychological side-effects of treatment. Information will also be given to patients on how they can contact the clinical team if they have concerns or symptoms; otherwise, they will receive routine telephone contact 6 months later. The telephone follow-up will involve a structured interview whereby patients’ unmet needs will be identified and patients will receive appropriate referral(s). The new approach will maintain regular contact with patients but will not involve routine hospital, outpatient attendance. The aim of this follow-up programme is to manage any long-term effects from treatment, to reassure the patient that the cancer remains in remission and if relapse occurs, to detect and treat it early.

This protocol falls within a larger body of work which includes a national survey relating to current practice of follow-up for gynaecological cancer patients in the UK [30], a BCUHB wide audit (headed by SL) of gynaecological cancer patients to establish key parameters in relation to recurrence and a small feasibility trial of a short-form needs assessment tool developed by one co-author (VM) and colleagues. Funding from Tenovus Cancer Care has been received for a Ph.D. student (LT, supervised by RTE, NS, VM) to explore work relating to patient and caregivers’ perspectives and preferences for different models of follow-up care [34]. Finally, we are currently conducting a systematic literature review to provide an up-to-date evaluation of the available evidence for the different strategies of follow-up after endometrial and vulval cancer. This body of work aims to show the wide applicability of the proposed intervention, whilst using this study as an exemplar of the methodology.

This study will focus on gynaecological cancer but will link with similar studies being developed in North Wales that will look at other exemplars, particularly prostate cancer (TOPCAT-P; ISRCTN34516019). The studies will be independent but will share expertise and co-investigators to develop a body of expertise relating the clinical research in this area.

## Methods/design

### Aim

The primary aim of the current study is to evaluate the feasibility of conducting a randomised trial comparing nurse-led telephone follow-up with standard, hospital-based, medical follow-up. Feasibility will be determined by the ability to recruit and retain patients to the study the ability to collect regular data and to evaluate the acceptability of implementing this new intervention.

The secondary aim of this feasibility study is to gain knowledge of standard deviations and effect sizes on the study outcome measures to inform the power calculation for a future full trial.

The final aim is to pilot the statistical and health economics analysis that would be required within a future full trial that will ultimately determine the effectiveness and cost-effectiveness of the new nurse-led telephone follow-up approach.

### Design

The current study will be a parallel-group randomised controlled feasibility trial design comparing nurse-led intervention (treatment group) with standard care (control group) to assess recruitment and retention rates and acceptability of randomisation and to inform the sample size calculation for a future trial.

### Participants

#### Inclusion criteria

We propose to recruit a cohort of female patients who have completed treatment for cervical, endometrial, epithelial ovarian, or vulval cancer within the last 3 months in BCUHB in North Wales. Patients who have completed treatment for fallopian tube and primary peritoneal carcinoma will also be included. The cohort will be identified through the regional gynaecological cancer multidisciplinary team meeting (MDT) records and will consist of patients who are considered fit for taking part in the trial and able to give informed consent, as assessed by the MDT. Patients may have received surgery, chemotherapy, radiotherapy or a combination of these. At the time of entry, patients in the view of their treating consultant will not have a definite need for continued hospital-based care.

#### Exclusion criteria

Patients having had treatment for sarcoma, germ cell tumour, borderline tumours or choriocarcinoma will be excluded as these women tend to require specific and/or more intense follow-up often with serial imaging or tumour markers. Patients requiring ongoing treatment will also not be included in the study. The study will not include patients who do not have the capacity to give informed consent or who are deemed to be unable to take part in the trial (e.g. severe learning/mental disability, severe mental health problems). Patients who are not able to understand Welsh or English will also be excluded.

### Sample size

We estimate 30% will not fit the inclusion criteria whilst an unknown proportion of the remainder will not enter the study, either through not wishing to consent or for other reasons. The extent of patient acceptability is unknown but for planning purposes, we will assume 50% acceptance. Assuming a throughput of approximately 150 newly diagnosed cases, we hope to randomise 50 participants from the recruiting centres into the study during a period of 6 months. It is thought this would be sufficient to inform a sample size of a future full trial [35] and enable a reasonably diverse group to fully assess the acceptability of the trial.

### Recruitment

All patients will be recruited from three hospitals in the North Wales Cancer Network: Ysbyty Gwynedd, Ysbyty Glan Clwyd, and Ysbyty Maelor. The research nurse and three clinical nurse specialists (CNSs) will identify women from the hospital databases, in line with the inclusion and exclusion criteria above. Eligible patients will be approached by the clinic medical team and/or by the research nurse/CNS and will be invited to take part in the study. Patients will be given a participant information sheet at their end-of-treatment visit and will have until their follow-up appointment to consider the study (usually around 6 weeks), and those who would like to participate will have the opportunity to contact the research nurse by phone or email for more information before their follow-up appointment. Patients will be informed that if they agree to participate, they will be randomly assigned to one of the two treatment groups, both receiving slightly different modes of follow-up support. After agreeing to take part in the study, patients will give written consent at their 3-month routine follow-up appointment.

### Randomisation

Following their 3-month appointment, consenting patients will complete the baseline measures (see below) then be randomised at the end of the 3-month review appointment. Patients will be randomised on a 1:1 ratio by the North Wales Organisation for Randomised Trials in Health (NWORTH) using a dynamic, independent, secure, web-based, randomisation procedure that can be accessed 24 h a day [36]. Patients will be randomised to one of the two arms of the study: standard care or nurse-led intervention. Site and disease type will be included as stratification variables. The participants, research nurse, CNSs and trial management will be unblinded during this trial. All other members of the team, especially the trial statistician, will remain blinded.

### Standard care

After patients have been allocated to the standard care arm of the study, they will be informed about the next part of the study and their 6- and 9-month appointments scheduled. Patients randomised to standard care will continue to have their usual hospital-based doctor-led medical reviews (at 6 and 9 months post-treatment conclusion) and will be followed up according to an agreed protocol with the regional gynaecological cancer MDT and representing current practice. This will include an agreed protocol on further blood tests and scans if considered appropriate to routine follow-up.

In addition to their usual follow-up appointments, patients will be asked to complete a set of outcome questionnaires at baseline (following consent, but prior to randomisation), with assistance from the research nurse or CNS at the 3-month follow-up appointment. Patients will then be asked to complete the same set of outcome measures at the end of each of their follow-up appointments. If this is not possible, then the research nurse will send the questionnaires by post for completion and return in a self-addressed, pre-stamped envelope.

### Nurse-led intervention

Patients in the intervention group will not attend the hospital for their usual follow-up appointments but will receive a nurse-led telephone follow-up intervention, known as Optimal Personalised Care After Treatment for Gynaecological cancer (OPCAT-G). After patients have been allocated to the nurse-led intervention arm of the study, the research nurse or CNS will inform patients of their allocation. The CNS will give the patients an information booklet at the end of their 3-month medical review and will guide the patient through the sections of the booklet. The information booklet will include information on patterns of relapse, possible warning symptoms and how to respond to these. Information will also be given on possible long-term physical and psychological side-effects of treatment and how these can be managed. Information will also be given to patients on how they can contact the clinical team if they have concerns or symptoms. Patients will receive treatment and diagnosis-specific supplementary leaflets attached to the booklet. The CNS will also give patients a set of needs assessment measures, including:

- Macmillan Concerns Checklist [37]
  The Macmillan Concerns Checklist addresses 23 physical concerns; 9 practical concerns; 3 family/relationship concerns; 9 emotional concerns; 3 spiritual or religious concerns; and 9 lifestyle or information needs. Patients are asked to tick a box for any problems that have caused concern during the previous week or to leave the box blank if it does not apply to them.
- CancerCAN-22 [24, 38, 39]
  This is a multi-domain needs assessment tool that measures unmet needs across 22 items (12 psychosocial needs items and 10 treatment and care items). Items are either met, unmet or never had/not applicable; then scored for salience if unmet on a 0–3 Likert scale (no importance, low importance, medium importance and high importance).
- Distress thermometer
  This is a reliable measure of patients’ distress and concerns and is widely used in cancer studies [40]. It has accepted cut-offs indicative of clinical levels of global distress.

Patients will be asked to complete the needs assessment measures prior to a scheduled telephone call from the CNS. Patients will receive a scheduled telephone call within 4 weeks of randomisation and will be notified of their scheduled telephone call date and time at the end of their 3-month medical review. Patients will also receive a letter confirming the date and time of their scheduled telephone call.

The scheduled telephone call will involve a structured interview with the CNS. Patients will be asked about their general wellbeing and about any gynaecological symptoms experienced. Any identified needs or concerns from the three needs assessment measures will be discussed. Patients in whom problems or unmet needs are identified will be evaluated and directed to the most appropriate source of help. This could include a patient self-help group, their general practitioner, or hospital review by the gynaecology, oncology or clinical psychology team. These additional appointments would be facilitated by the CNS who would ensure appropriate communication regarding the reasons for referral. This information, including advice and follow-up suggestions, will be recorded using a case note sheet. Patients referred for further hospital review will be discharged to continue telephone review once their immediate problem has been assessed and treated or resolved. Patients will receive additional copies of the needs assessment measures by post. Patients will be told that if they have any problems between telephone calls, they should complete their assessments and contact the CNS. Patients will be encouraged to report problems promptly with these concerns addressed in the same structured way.

Patients will then receive another scheduled telephone call at 9 months post-treatment. Patients will receive a letter confirming the date and time of their telephone call appointment by post. Patients will also receive the three needs assessment measures by post and will be asked to complete them prior to their scheduled telephone call from the nurse. The telephone contact will again include a structured interview and any identified needs or concerns will be discussed following the same structure of the first telephone call interview.

In addition to the nurse-led intervention, patients will be asked to complete the same set of outcome questionnaires as patients in the standard care arm. The outcome questionnaires will be completed at baseline (prior to randomisation) with assistance from the research nurse or CNS at the 3-month follow-up appointment. Patients will receive the same set of outcome questionnaires by post at 6 months post-treatment and within a week of their 9-month telephone follow-up and will be asked to return them in a self-addressed, pre-stamped envelope.

For this study, we will consider the study endpoint to be the 9 months post-treatment data collection. After the 9-month data collection point, the intervention and control arm patients will continue with follow-up care as the clinician advises and is available to the local service.

### CONSORT Diagram

**Fig. 1:**
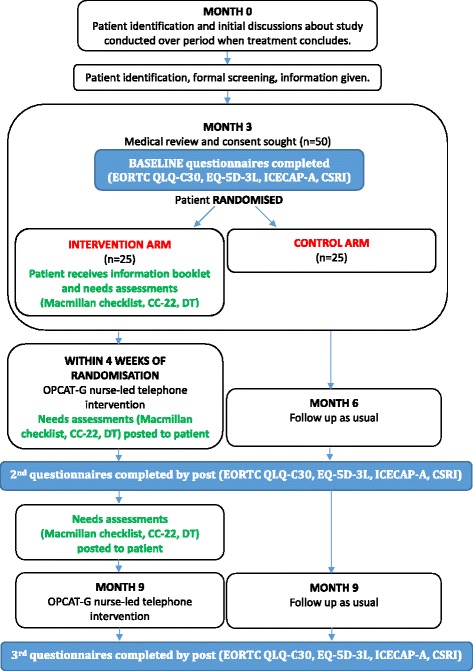
CONSORT Diagram for TOPCAT-G feasibility study

**Fig. 2:**
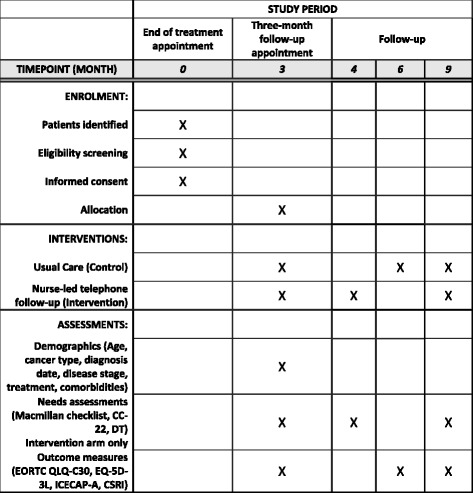
CONSORT Diagram for TOPCAT-G feasibility study

The CONSORT Diagram, which will be used to illustrate recruitment and loss during the study, can be found in Fig. 1. For a further detailed breakdown of the patient involvement in the study, Fig. 2 shows the SPIRIT flowchart of the trial.

### Measures

#### Primary outcome measures

The primary outcome measures of the feasibility trial will be:

- Patient recruitment rate
  This will be calculated from the total number of eligible patients invited to take part in the study and the number of patients giving written consent to participate in the study.
- Patient attrition rate
  This will be calculated from the number of patients who gave written consent and the number of patients who have completed any measures, regardless of their completion rate.

#### Secondary outcome measures

A single booklet of outcome questionnaires will be provided to all trial participants at baseline and 6 and 9 months post-treatment. These are as follows:

- EORTC QLQ-C30
  This is a validated measure to assess the quality of life of cancer patients [41]. It comprises a 30-item questionnaire incorporating five functional scales (physical, role, cognitive, emotional and social), three symptom scales (fatigue, pain and nausea and vomiting), a global health status/quality of life scale and a number of single items assessing additional symptoms commonly reported by cancer patients and perceived financial impact of the disease.
- EQ-5D-3L
  This is a validated generic, health-related, preference-based measure comprising five domains: mobility, self-care, usual activities, pain and discomfort and anxiety and depression. Each domain has three levels (no problems, some problems and a lot of problems). The EQ-5D-3L scoring system defines 243 possible health states. The questions are complemented by a visual analogue scale (VAS), on which respondents are asked to indicate their current health [42].
- ICECAP-A (ICEpop CAPability measure for Adults)
  This is a measure of capability for the general adult population [43]. The ICECAP-A covers five attributes of wellbeing: attachment, stability, achievement, enjoyment and autonomy.
- Client Service Receipt Inventory (CSRI)
  This is an adapted version of a standardised measure using self-report service user data to evaluate and cost service use, including all GP visits and unscheduled secondary care in both arms of the study [44, 45].

Additional data to be captured will include the following:

e) Patient demographics, cancer type and stage, type of treatment received and co-morbidity at baseline (from medical records and patient confirmation).
f) Data on the pattern, timing and method of detection of relapse and on survival (recorded using a Serious Adverse Events form).

We anticipate that on average patients would complete all questionnaires in about 20 min on each occasion.

The number of patients offered the study will be logged to identify the proportion of patients that decline to take part. Patients who decline to consent will be asked if they would be willing to provide any reason(s) why they preferred not to take part.

Data will aim to be collected from all willing participants, even in the case of a withdrawal or non-compliance with the intervention.

### Data collection/storage

Data collection, cleaning and analysis will be undertaken by NWORTH using standard, secure, anonymous procedures for handling patient data. The fully auditable MACRO data management system will be used to ensure best practice. The health economics and patients’ demographic data will be transferred from NWORTH to CHEME (Centre for Health Economics and Medicines Evaluation) for further analysis.

### Statistical methods

Descriptive statistics will be reported, including clinical and demographic characteristics of those recruited. To assess the feasibility of a full RCT, the recruitment and retention rates will be calculated. A future full RCT will be considered feasible if the recruitment rate is at least 50% and retention rate is no less than 50%. Additionally, the patient eligibility rate and questionnaire completion rates will be calculated to provide further evidence for the appropriateness of a full trial and associated measures.

Exploratory analysis will also be conducted around the assessments of QoL with the purpose of estimating key parameters for powering the main RCT but not to provide definitive results for the study. Effect sizes of the differences in final scores calculated from the EORTC QLQ-C30 will be calculated between the groups as randomised for the study endpoint. The effect size will be used to inform future sample size calculations for a larger, multi-centre RCT.

### Health economics

An exploratory health economic analysis will be conducted to assess the feasibility of answering the required research questions within a full RCT, but not to provide definitive results for the study at this stage. Descriptive statistics will be performed to assess the responses of the self-reported health service use, EQ-5D-3L and ICECAP-A and the level of completeness for EQ-5D-3L and ICECAP-A in both arms of the study; this will allow us to explore as to how well the self-administered questionnaires perform in this patient population group. The strength of the relationship between EQ-5D-3L index score and ICECAP-A score will be explored using Pearson’s Product Moment Correlation Coefficient. An exploratory cost-effectiveness analysis will be undertaken from a multi-agency perspective to explore the cost-effectiveness of a ‘nurse-led’ intervention compared to standard care; this will help power economic analysis alongside a future full trial.

### Trial management plan

A trial working group (WG) consisting of individuals responsible for the effective day-to-day running of the trial will meet on a monthly basis. The WG will be chaired by the chief investigator (CI). The WG will report regularly to the steering group (SG) to highlight any areas of difficulty.

The trial will be managed by a SG, chaired by one of the co-applicants. The SG will meet at least three times per year to oversee the overall running of the trial and to ensure timely progress with trial development, implementation and reporting. It will comprise the CI and co-applicants.

Two patient representatives from the North Wales Cancer Forum Group will be invited to attend SG meetings in order to contribute to discussions regarding study recruitment methods and materials, including the patient information sheets and consent materials and the intervention protocol. This group will also benefit the project team in terms of advising as to the appropriate interpretation and dissemination of findings and to how best take forward the findings into development of the ensuing RCT protocol.

A Data Monitoring and Ethics Committee (DMEC) will independently monitor the data and quality assurance of the study. This will consists of an independent chair, statistician and expert in the field.

Day-to-day running and co-ordination of the trial will be undertaken by an appropriately experienced research officer employed by NWORTH. This work will be supported by the research nurse, a new appointment arising from the collaboration between Health and Care Research Wales and BCUHB to support the portfolio of such studies within BCUHB. Both organisations have well-developed research governance procedures and have joint standard operating procedures (SOPs) for research. All research staff will receive Good Clinical Practice training, SOP training and any further task-specific training identified. Full training details will be maintained in individual staff training matrices as per SOP.

### Reports and dissemination

Findings from this study will be presented via posters and oral presentations at regional and national meetings where oncology doctors, specialist nurses and health service managers would be present. This would include specialist meetings relating to gynaecological oncology, medical oncology, health psychology/psycho-oncology and health economics.

Papers will be submitted to relevant international journals such as International Journal of Gynecological Oncology, British Journal of Obstetrics and Gynaecology, Lancet Oncology and British Journal of Cancer.

Preparation of the protocol for a national randomised, multi-centre trial comparing OPCAT-G with standard care would be a key output from this work. Publications are also expected from the work of the Tenovus Ph.D. student regarding patients, carers and health care professionals preferences for gynaecological cancer follow-up care.

## Discussion

The TOPCAT-G study will evaluate the feasibility of completing a full, definitive RCT that would assess the potential benefits of nurse-led telephone follow-up for gynaecological cancer patients compared with current follow-up care comprised of regular outpatient appointments. Feasibility for the study will be judged using patient recruitment and attrition rates. The study will also provide indications on the suitability of the outcome measures and will pilot the proposed statistical analysis plan and also a cost-effectiveness analysis plan which would be an essential part of a full RCT. The results found from the feasibility study will also be used to inform the sample size of a future RCT.

A potential operational issue for the study might be in finding an appropriate space for each of the CNSs to complete their telephone follow-ups with the participants. Gaining information on issues such as this will be a vital part of shaping the design of a future RCT. An unknown part of the study is the amount of time each of the telephone follow-ups will take for the CNSs to complete; this will be collected and collated at the end of the feasibility study to help more accurately inform a future RCT.

### Trial status

The TOPCAT-G study started in July 2014, screening began in September 2015 giving a recruitment period of November 2015 to the end of April 2016. Follow-ups will continue until October 2016, and the trial is due to end in January 2017.

## Abbreviations

BCUHB: Betsi Cadwaladr University Health Board
CHEME: Centre for Health Economics and Medicines Evaluation
CI: Chief Investigator
CNS: Clinical nurse specialist
CSRI: Client Service Receipt Inventory
EQ-5D-3L: EuroQoL-5 dimensions-3 levels
GP: General Practitioner
HR-QoL: Health-related quality of Life
ICECAP-A: ICEpop CAPability measure for Adults
MDT: Multidisciplinary team meeting
NHS: National Health Service
NICE: The National Institute for Health and Care Excellence
NISCHR CRC: National Institute for Social Care and Health Research Clinical Research Centre
NSAG: National Strategic Advisory Group
NWORTH: North Wales Organisation for Randomised Trials in Health
QoL: Quality of life
R&D: Research and Development
RCT: Randomised controlled trial
SG: Steering group
SOP: Standard operating procedure
TOPCAT-G: Trial of Optimal Personalised Care After Treatment for Gynaecological cancer
TOPCAT-P: Trial of Optimal Personalised Care After Treatment for Prostate cancer
VAS: Visual analogue scale
WG: Working group
